# Experimental Oral Transmission of Chronic Wasting Disease to Reindeer (*Rangifer tarandus tarandus*)

**DOI:** 10.1371/journal.pone.0039055

**Published:** 2012-06-18

**Authors:** Gordon B. Mitchell, Christina J. Sigurdson, Katherine I. O’Rourke, James Algire, Noel P. Harrington, Ines Walther, Terry R. Spraker, Aru Balachandran

**Affiliations:** 1 National and OIE Reference Laboratory for Scrapie and CWD, Canadian Food Inspection Agency, Ottawa Laboratory – Fallowfield, Ottawa, Ontario, Canada; 2 Departments of Pathology and Medicine, University of California, San Diego, La Jolla, California, United States of America; 3 Department of Pathology, Microbiology and Immunology, University of California, Davis, California, United States of America; 4 Animal Disease Research Unit, Agricultural Research Service, United States Department of Agriculture, Pullman, Washington, United States of America; 5 Veterinary Diagnostic Laboratory, Colorado State University, Fort Collins, Colorado, United States of America; Creighton University, United States of America

## Abstract

Chronic wasting disease (CWD), a transmissible spongiform encephalopathy of cervids, remains prevalent in North American elk, white-tailed deer and mule deer. A natural case of CWD in reindeer (*Rangifer tarandus tarandus*) has not been reported despite potential habitat overlap with CWD-infected deer or elk herds. This study investigates the experimental transmission of CWD from elk or white-tailed deer to reindeer by the oral route of inoculation. Ante-mortem testing of the three reindeer exposed to CWD from white-tailed deer identified the accumulation of pathological PrP (PrP^CWD^) in the recto-anal mucosa associated lymphoid tissue (RAMALT) of two reindeer at 13.4 months post-inoculation. Terminal CWD occurred in the two RAMALT-positive reindeer at 18.5 and 20 months post-inoculation while one other reindeer in the white-tailed deer CWD inoculum group and none of the 3 reindeer exposed to elk CWD developed disease. Tissue distribution analysis of PrP^CWD^ in CWD-affected reindeer revealed widespread deposition in central and peripheral nervous systems, lymphoreticular tissues, the gastrointestinal tract, neuroendocrine tissues and cardiac muscle. Analysis of prion protein gene (*PRNP*) sequences in the 6 reindeer identified polymorphisms at residues 2 (V/M), 129 (G/S), 138 (S/N) and 169 (V/M). These findings demonstrate that (i) a sub-population of reindeer are susceptible to CWD by oral inoculation implicating the potential for transmission to other *Rangifer* species, and (ii) certain reindeer *PRNP* polymorphisms may be protective against CWD infection.

## Introduction

Chronic wasting disease (CWD) is an invariably fatal neurodegenerative disease of cervids belonging to the transmissible spongiform encephalopathy (TSE) group of disorders which include Creutzfeldt-Jakob disease (CJD) in humans, bovine spongiform encephalopathy (BSE) in cattle and scrapie in sheep and goats. Common among TSEs is a pathogenesis contingent on the conversion of a normal host-encoded prion protein (PrP^C^) to an abnormal disease-associated isoform (PrP^CWD^) which accumulates in the nervous system and other tissues, resulting in the progressive manifestation of clinical disease. CWD is the only TSE maintained in free-ranging wildlife and the relatively efficient horizontal transmissibility of CWD between conspecific cervids presumably relates to the excretion of infectivity in saliva, urine and feces [1], [2] as well as its resiliency in contaminated environments [3], [4]. The transmissibility of CWD from cervids to domestic livestock, humans and other interacting wildlife species (e.g. carnivores) remains uncertain although current evidence suggests a considerable species barrier markedly impedes, if not prevents such transmission under natural circumstances.

Since the identification of CWD in captive deer in Colorado in 1967 [5], it has been described in captive and wild cervid populations in several US states, three Canadian provinces and Korea [6], [7], [8]. Naturally affected cervid species include mule deer (*Odocoileus hemionus*), white-tailed deer (*Odocoileus virginianus*), Rocky Mountain elk (*Cervus elaphus nelsoni*) and moose (*Alces alces*) [7]. Experimentally, CWD has been orally transmitted to red deer (*Cervus elaphus elaphus*) [9], with similar clinical and pathological findings to CWD in other cervids.

Chronic wasting disease in reindeer (*Rangifer tarandus tarandus*), or closely related caribou (e.g. *Rangifer tarandus caribou*, *Rangifer tarandus granti*, *Rangifer tarandus groenlandicus*) has not yet been reported despite the potential overlap in natural host ranges with CWD-susceptible cervids in North America. Determining transmissibility of CWD to reindeer and caribou is of particular importance given the cultural significance of these species to the aboriginal peoples of northern North America and Eurasia, the critically reduced populations of many reindeer and caribou herds, and the potential for some highly migratory herds to further disseminate CWD. Here we investigate the oral transmission of CWD from elk (CWD^ELK^) or white-tailed deer (CWD^WTD^) to reindeer and describe associated genetic, clinical and pathological findings.

## Results

### Antemortem Testing

**Figure 1:**
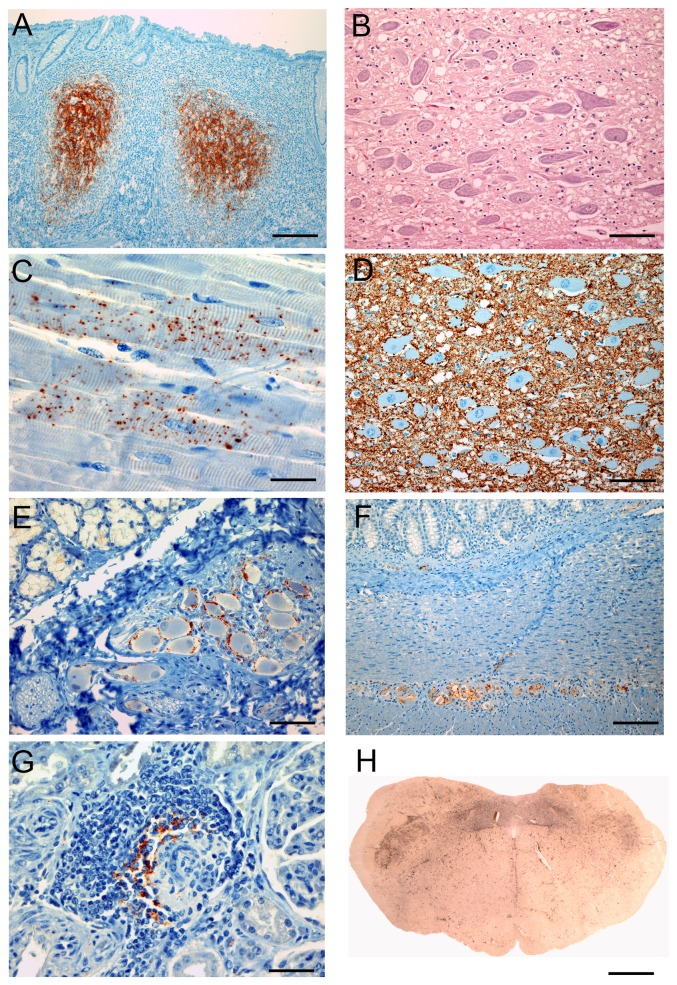
Tissue distribution of PrP^CWD^ and histopathology in clinically affected reindeer orally inoculated with CWD from white-tailed deer. IHC detection of PrP^CWD^ using MAb F99/97.6.1 demonstrates: (A) immunolabeling associated with tingible body macrophages and follicular dendritic cells in a RAMALT biopsy taken at 13.4 mpi, (C) punctate staining in cardiomyocytes, (D) widespread particulate deposits in the dorsal vagal nucleus, (E) immunolabeling within a ganglion adjacent to the submandibular salivary gland, (F) granular PrP^CWD^ in submucosal and myenteric plexuses of the ileum and (G) amongst glomerular lymphocytic infiltrates. (B) Haematoxylin and eosin stained section of the dorsal vagal nucleus reveals vacuolation of the neuropil. (H) PET blot of the obex demonstrating abundant widespread PrP^CWD^ filling the dorsal vagal nucleus and surrounding nuclei. Bars, 150 µm (A); 65 µm (B,D,E); 24 µm (C,G); 92 µm (F); 2.4 mm (H).

Animals were subjected to recto-anal mucosal lymphoid tissue (RAMALT) biopsy at various time points after inoculation to determine if they had been infected with CWD. Two of the 3 reindeer inoculated with CWD^WTD^ (Reindeer 12 and 47) showed marked accumulations of PrP^CWD^ in RAMALT germinal centers at 13.4 and 16.2 months post-inoculation (mpi)(Figure 1, Table 1). Testing of the biopsy material from month 16.2 by western blot and ELISA confirmed the presence of PrP^CWD^ in the same two animals with ELISA optical density (OD) values of 1.519 and 1.484 (negative OD cut-off  = 0.19). The 3^rd^ reindeer in the CWD^WTD^ inoculum group (Reindeer 17) was negative for PrP^CWD^ in all RAMALT samples by ELISA, western blot and IHC, with last sampling conducted at 25 mpi. Similarly, none of the reindeer inoculated with CWD^ELK^ (Reindeer 1, 2 and 60) showed detectable PrP^CWD^ in RAMALT biopsies at 7.5, 12, 17.5 or 54.8 mpi.

**Table 1:** Summary of results from reindeer orally inoculated with CWD from elk or white-tailed deer.^a^

ID	CWD Inoculum	Clinical Disease^b^	Survival Time (mpi)	*PRNP* Genotype^c^				IHC^d^		WB	ELISA^e^	
				2	129	138	169	RAMALT	Obex		Obex	LN
1	Elk	No	22.6	VV	GG	SN	VV	− (17.5)	−	−	−	−
2	Elk	No	60.9	VM	GS	SN	VM	− (54.8)	−	−	−	−
60	Elk	No	45.3	VM	GS	SN	VM	− (17.5)	−	−	−	−
12	WTD	18.2	20.0	VV	GG	SS	VV	+ (13.4)	+	+	+ (2.65)	+ (1.18)
47	WTD	17.7	18.5	VV	GG	SS	VV	+ (13.4)	+	+	+ (2.81)	+ (1.65)
17	WTD	No	NA^f^	VV	GG	SN	VV	− (25.0)	NA	NA	NA	NA

a ELISA  =  enzyme-linked immunosorbent assay; IHC  =  immunohistochemistry; LN  =  lymph node; mpi  =  months post-inoculation; NA  =  data not available; RAMALT  =  Recto-anal mucosa associated lymphoid tissue; WB  =  Western blot; WTD  =  White-tailed deer.

b Time (mpi) to first clinical signs of neurological disease if observed.

c Deduced amino acids at codons 2-129-138-169. V =  Valine; M =  Methionine; G =  Glycine; S =  Serine; N =  Asparagine.

d IHC conducted on RAMALT or obex tissue using mAb F99. The time of last RAMALT sampling is listed in parentheses (mpi) for animals with a negative IHC result, while the time of the first positive RAMALT biopsy is listed for the positive animals.

e Conducted on obex or retropharyngeal lymph node and reported as positive (+) or negative (–) with optical density (OD) in parentheses from a commercially available ELISA kit (negative OD cut-off  = 0.21).

f Animal 17 remains alive at 26 mpi with no evidence of clinical signs or PrP^CWD^ in RAMALT biopsy.

### Clinical Signs

Beginning around 17.7 to 18.2 mpi the two RAMALT-positive reindeer inoculated with CWD^WTD^ (Reindeer 12 and 47) began to show subtle weight loss and uneven hair coat. After briefly displaying ataxia, excessive salivation and grinding of the teeth both animals developed terminal CWD at 18.5 and 20 mpi. Other than markedly reduced subcutaneous, abdominal and thoracic adipose stores, gross lesions were not identified at necropsy. The third, RAMALT-negative reindeer (Reindeer 17) inoculated with CWD^WTD^ remains alive and has shown no clinical signs of CWD (currently @ 26 mpi). The reindeer receiving the CWD^ELK^ inoculum (Reindeer 1, 2 and 60) did not display clinical signs of CWD at any time during the study and were euthanized at 22.6, 45.3 and 60.9 mpi, respectively.

### Histopathology and Immunohistochemistry

Histopathological examination of brain tissues from both clinically affected reindeer revealed widespread spongiform pathology characterized by regionally extensive vacuolation of the grey matter neuropil and rare neuronal perikarya. Lesions were most prominent in the medulla, particularly the dorsal vagal nucleus (Figure 1), nucleus of the solitary tract, the reticular formation, cuneate nuclei and nucleus of the spinal tract of the trigeminal nerve. Notable spongiform change was also identified elsewhere in the brainstem, striatum, thalamus, cerebellum and to a lesser extent in the cerebral cortex.

Localization of PrP^CWD^ by IHC identified accumulations throughout the central and peripheral nervous systems, lymphoreticular tissues and neuroendocrine tissues of the two reindeer displaying clinical signs (Table 2, Figure 1). The tissue distribution and intensity of PrP^CWD^ accretion was generally consistent between both CWD-positive reindeer. Tissues listed in Table 2 were concurrently tested by a commercially available ELISA kit and results were found to corroborate IHC findings (data not shown). Conversely, PrP^CWD^-specific immunolabeling was not detected by IHC or ELISA in any of the tissues collected from the 3 non-clinical reindeer receiving the CWD^ELK^ inoculum.

**Table 2:** PrP^CWD^ distribution in tissues of reindeer orally inoculated with brain tissue from CWD-infected white-tailed deer.

System, anatomical site	Detection of PrP^CWDa^		System, anatomical site	Detection of PrP^CWDa^
**Central nervous system**			**Lymphoreticular system**	
Frontal cortex	+		RAMALT	+
Basal ganglia	+		Nictitating membrane	+
Thalamus	+		Palatine tonsil	+
Midbrain	+		Retropharyngeal LN	+
Rostral medulla	+		Parotid LN	+
Obex	+		Submandibular LN	+
Cerebellum	+		Pre-scapular LN	+
Spinal cord (C5, T6, L5)	+		Mediastinal LN	+
**Peripheral nervous system**			Tracheobronchial LN	+
Trigeminal ganglia	+		Hepatic LN	+
Celiac ganglia	+		Rumenal LN	+
Vagus nerve	+		Abomasal LN	+
Phrenic nerve	−		Mesenteric LN	+
Sympathetic trunk	+		Ileocecal LN	+
Sciatic nerve	−		Sublumbar lN	+
**Gastrointestinal system**			Pre-femoral LN	+
Submandibular salivary gland	+		Popliteal LN	+
Parotid salivary gland	−		Spleen	+
Esophagus	+		**Musculoskeletal system**	
Rumen	+		Tongue	−
Reticulum	+		Masseter	−
Abomasum	+		Diaphragm	−
Omasum	+		Trapezius	−
Duodenum	+		Triceps brachii	−
Jejunum	+		Semitendinosus	−
Ileum	+		Psoas major	−
Ceacum	+		**Other tissues**	
Colon	+		Heart	+
**Endocrine system**			Lung	+
Pancreas	+		Nasal mucosa	+
Adrenal gland	+		Liver	−
Thyroid gland	+		Kidney	+
Pituitary gland	+		Bladder	+

a PrP^CWD^ was detected by IHC with MAb F99/97.6.1 and a commercially available TSE ELISA kit and results are expressed as PrP^CWD^ present (+) or not detected (−).

RAMALT, recto-anal mucosa-associated lymphoid tissue; LN, lymph node.

Consistent with the distribution of spongiform pathology, PrP^CWD^ was distributed throughout the brain with highest intensity in the medulla and relatively lower deposition in the cerebral cortex. The most abundant type of PrP^CWD^ accumulation in the central nervous system was fine particulate, accompanied by coarse granular and coalescing deposits in heavily affected regions. Throughout sections at all levels of spinal cord, particulate and granular PrP^CWD^ accumulations were present in the dorsal, lateral and ventral horns, scattered lightly around white matter and within perikarya of the dorsal root ganglia. In the eye, PrP^CWD^ was most prominent in outer and inner plexiform layers of the retina with a scant presence in the ganglion cell layer and optic nerve.

In the peripheral nervous system, occasional perineuronal PrP^CWD^ was found in trigeminal and celiac ganglia. Scant, coarse granular PrP^CWD^ was identified along nerve fibers and in association with perikarya of the sympathetic trunk, while sciatic and phrenic nerves did not contain detectable PrP^CWD^. Occasional granular PrP^CWD^ was present within nerve fascicles of the vagus nerve. Prominent accumulations of PrP^CWD^ were consistently affiliated with neurons of the submucosal and myenteric plexuses throughout the gastrointestinal tract and in small ganglia associated with the submandibular salivary gland and bladder.

All tissues of the lymphoreticular system, regardless of their proximity to the gastrointestinal tract, displayed particularly intense PrP^CWD^ accumulations in association with lymphoid follicle germinal centers. The percentage of follicles affected in each lymph node or tonsil was typically between 90 and 100 percent. In sections of kidney from one animal, two glomeruli were found to be affected by mild lymphocytic infiltrates which stained positive for PrP^CWD^. Similarly, rare lymphoid aggregates within lung tissue were associated with modest PrP^CWD^ deposits.

Several neuroendocrine tissues also contained abundant PrP^CWD^ deposits. Coarse granular staining within the pancreas was restricted to the islets of Langerhans and deposits in the adrenal gland were predominantly confined to the medulla. The pituitary gland contained heavy PrP^CWD^ staining in the pars nervosa and intermedia while rare granular staining was found in association with parafollicular cells of the thyroid gland. Thorough examination of several skeletal muscle groups by IHC did not reveal PrP^CWD^, however, sections of the ventricular myocardium were found to contain scattered foci of punctate granular staining in cardiomyocytes.

### Western Immunoblot

**Figure 2:**
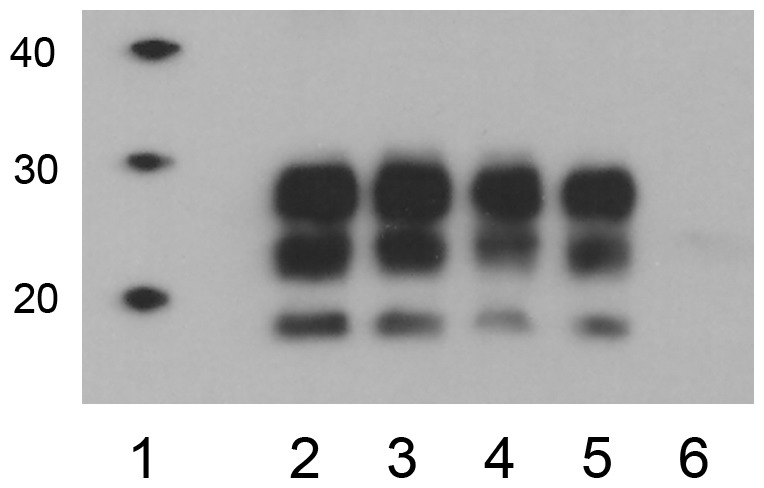
Western immunoblot of PrP^res^ in the brainstem of reindeer compared to elk and white-tailed deer CWD inocula. Reindeer 12 (lane 4) and reindeer 47 (lane 5) developed terminal CWD at 18.5 and 20 mpi. Lane 1– molecular weight marker (kDa), lane 2– CWD in elk, lane 3– CWD in white-tailed deer, lane 4– CWD in reindeer 12, lane 5– CWD in reindeer 47, lane 6– negative control elk.

Analysis of medulla oblongata samples from CWD^WTD^-inoculated reindeer by western immunoblot demonstrated proteinase K-resistant PrP (PrP^res^) in the 2 clinically affected animals (Figure 2) but not in the 3 animals inoculated with CWD^ELK^ (data not shown). The PrP^res^ glycosylation patterns (di-, mono- and unglycosylated bands) observed in the clinical reindeer were comparable in molecular weight and glycoform ratio to that of the original white-tailed deer inoculum.

### *PRNP* Sequence Analysis

**Figure 3:**
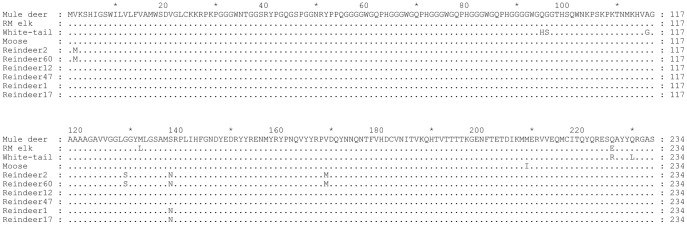
*PRNP* sequence of the reindeer used in this study compared with mule deer, white-tailed deer, Rocky Mountain elk, and moose. Reindeer are polymorphic at codons 2 (M/V), 129 (S/G), 138 (S/N), and 169 (M/V). *PRNP* polymorphisms have also been reported in Rocky Mountain elk (132 M/L), mule deer (20 D/G, 225 S/F), white-tailed deer (95 Q/H, 96 G/S, 116 A/G, 226 Q/R, and 230 Q/L) and moose 209 (M/I) [11], [41], [44], [45], [46], [47], [48].

Sequencing of the prion protein coding region (*PRNP*) revealed some variability among the 6 reindeer. The *PRNP* from reindeer 12 and 47 encoded a PrP amino acid sequence identical to that of mule deer (Figure 3), and both reindeer were susceptible to infection with CWD^WTD^. In contrast, *PRNP* from reindeer 2 and 60 were heterozygous at bases 4 (codon 2, V/M), 385 (codon 129, G/S), 413 (codon 138, S/N), and 505 (codon 169, V/M) of *PRNP* (cervid numbering), and completely resisted infection with CWD^ELK^ up to 60 mpi (Table 1). Finally, reindeer 1 and 17 were heterozygous at base 413 (codon 138, S/N) of *PRNP*. Reindeer 1 was euthanized at 22.6 mpi with no evidence of PrP^CWD^ in any tissue. Reindeer 17 is still alive and without clinical signs at 26 mpi and thus far has no PrP^CWD^ in lymphoid tissue, implicating 138 N as a protective polymorphism. All reindeer were methionine homozygous at codon 132 of the cervid prion protein, which corresponds to polymorphic codon 129 in humans.

## Discussion

The current study demonstrates the oral transmission of CWD from the brain tissues of infected white-tailed deer to reindeer (*Rangifer tarandus tarandus*). Antemortem testing identified PrP^CWD^ in two reindeer at 13.4 mpi and both animals manifested consistent clinical symptoms and succumbed to disease by 20 months. The course of disease progression and widespread distribution of PrP^CWD^ in nervous and lymphoreticular systems are consistent with observations from other cervids naturally or experimentally infected with CWD, including elk, white-tailed deer, mule deer, moose and red deer [9], [10], [11]. A natural case of CWD in reindeer or caribou has not yet been identified but given the potential overlap in habitat with infected free-ranging cervid populations and the current findings of oral transmissibility, the potential for natural transmission certainly exists.

Cervids with terminal CWD typically display PrP^CWD^ deposition throughout multiple organ systems which progresses from early lymphatic tissue involvement, to central and peripheral nervous tissues followed by accumulation in other tissues including the endocrine system and heart [12], [13]. In our study, RAMALT sampling prior to 13.4 mpi was not conducted precluding an accurate determination of when lymphoid involvement occurred, although PrP^CWD^ has been detected in peripheral lymphoid tissue as early as 42 d after oral inoculation of mule deer fawns [14]. Our observations of PrP^CWD^-specific staining in sections of pancreas, pituitary, adrenal medulla, lung, kidney, bladder and salivary gland corroborate findings in other CWD-infected cervids [9], [12], [13], with PrP^CWD^ primarily localized to lymphoid or neural components of these tissues. The presence of PrP^CWD^ in reindeer excreta such as saliva, urine and feces is anticipated based on studies in deer [1], [2] but was not investigated here. Skeletal muscle from CWD-infected mule deer has been shown to contain infectivity [15] and PrP^CWD^ was recently reported in muscle-associated nerve fascicles of white-tailed deer [16]. We were unable to detect PrP^CWD^ in myocytes or associated nerves by IHC or ELISA consistent with the negative findings of others [9], [12], [17], [18]. Regardless, the widespread distribution of PrP^CWD^ in infected reindeer tissues merits consideration while evaluating the risks emanating from processing or consuming potentially infected animals.

None of the reindeer inoculated with CWD^ELK^ developed clinical disease or accumulated PrP^CWD^ in any of the tissues tested. The apparent resistance of reindeer to infection by the CWD^ELK^ inoculum may relate to subtle differences in the CWD strains present within the pooled elk and white-tailed deer inocula, however, we think this is unlikely. Differences between the two inocula were not evident through glycosylation profile analysis of PrP^res^ by western blot (data not shown). Further characterization of these inocula in transgenic mice is ongoing. We also considered that this may have been due to a low prion titre in the elk inoculum, however, the same CWD inoculum pool has been used in other studies and was highly infectious for red deer [9] and elk [19]. Alternatively, resistance could be due to the *PRNP* genotype, in which heterozygous codons occurred at positions 2 (V/M), 129 (G/S), 138 (S/N), and 169 (V/M) in the mature form of PrP^C^. An important role for *PRNP* polymorphisms in determining disease susceptibility is also suggested by the finding that one of the three reindeer has not yet succumbed to infection with the CWD^WTD^ inoculum by 26 mpi and showed no detectable PrP^CWD^ in lymphoid tissue at 25 mpi. This possibly resistant reindeer differs from the susceptible reindeer in being heterozygous at a single codon 138 (S/N).

The role of codons 2, 129 and 169 in CWD resistance is unknown. Interestingly, 138 N is common to the 4 reindeer that resisted elk or white-tailed deer CWD infection and may offer some protection. Consistent with this possibility, fallow deer were reported to express a *PRNP* gene encoding 138 N and 226 E and resisted infection when housed together with CWD-infected mule deer for 7 years [20]. Residue 138 N was implicated as the residue possibly protective against CWD infection, since red deer that express PrP^C^ with 226 EE are highly susceptible to elk CWD infection [9]. Fallow deer were shown to be susceptible to CWD inoculation by the intracerebral route with long incubation periods of 4–5 years [21]. Nevertheless, it will be important to determine whether fallow deer resist CWD after an oral challenge.

Codon 138 is in the β1−α1 loop region [22]. Although serine and asparagine are similar small, polar residues, serine/asparagine differences at position 173 (elk numbering) of the β2−α2 loop may impact the susceptibility of a species to CWD infection [23], [24]. Additionally, crystal structures of the β2−α2 loop peptides have shown that 173 (S/N) and 177 (N/T) sequence differences modify how the side chains pack at the β-sheet interface, and suggest that this incompatibility may underlie transmission barriers [25]. Whether serine or asparagine at position 138 has a similar effect on β-sheet packing is not yet known.

Would the reindeer that were heterozygous at 2 (V/M), 129 (G/S), 138 (S/N), and 169 (V/M) completely resist CWD infection or require a long incubation period to develop PrP^CWD^ or clinical signs of disease? No trace of PrP^CWD^ in lymphoid tissue or any other tissues at the end of the experiment (60 mpi) suggests at minimum a long delay in developing CWD infection, and at best complete protection from CWD infection.

In North America, the term reindeer describes a domesticated *Rangifer tarandus* subspecies (*Rangifer tarandus tarandus*) introduced from Eurasia approximately a century ago, while the term caribou refers to several native wild *Rangifer tarandus* subspecies. Major caribou subspecies include woodland (*Rangifer tarandus caribou*) and barren-ground (*Rangifer tarandus groenlandicus*, *Rangifer tarandus granti*) caribou [26]. Barren-ground caribou generally exist in higher northern latitudes, in some regions commingling with semi-domesticated reindeer herds, while the range of woodland caribou extends further south into the provinces of Alberta and Saskatchewan which currently harbour CWD-infected cervids. A study of 95 caribou in northern Quebec failed to identify CWD [27], although CWD has not yet been detected in Canadian provinces east of Saskatchewan.

Interbreeding between reindeer and caribou has occurred in captivity and the wild although general genetic introgression appears to be minimal [28]. Two of the reindeer protected from CWD were heterozygous at 4 codons that were in common with the reported genotypes of *Rangifer tarandus granti* [29] and differ from *Rangifer tarandus tarandus* (unpublished, Genbank accession #AY639093.01). It is possible that the two resistant reindeer were derived from interbreeding two *Rangifer tarandus* subspecies, potentially captive and free-ranging. It will be crucial to determine the prevalence of the polymorphisms in both free-ranging and captive reindeer populations, particularly considering the possible protective *PRNP* residues.

Large scale analysis of *PRNP* alleles in reindeer has not been reported. Largely consistent with our findings in captive reindeer, Happ *et al*. [29] recently sequenced *PRNP* from three free-ranging caribou herds in Alaska and identified polymorphisms at codons 2 (V/M), 129 (G/S), 138 (S/N) and 169 (V/M). Moreover, the most frequent caribou allele across the 3 herds examined was VGSV, which is common to the 2 reindeer we found to be susceptible to CWD^WTD^. The close interrelatedness of *Rangifer* species implies disease susceptibility in one may predict susceptibility in the others, although a single amino acid difference in a key position could confer resistance.

The importance of *Rangifer* species to the culture of aboriginal peoples cannot be underestimated with many components of hunted animals being consumed as food. Although relatively limited in comparison to elk and deer industries in North America, reindeer and caribou farming does occur, producing consumables such as meat, hides and antler velvet. The human health risks of consuming meat or other products derived from CWD-infected animals remain uncertain, although epidemiological evidence indicates transmission has not yet occurred [30], [31], [32] and transgenic mouse studies suggest the risk is remote in humans expressing common *PRNP* sequences [33], [34], [35]. The finding that CWD can be transmitted to squirrel monkeys by intracranial inoculation [36] raises concern for human transmissibility, however a study in macaques failed to demonstrate transmission after 70 months [37]. Since prion strains may undergo changes in transmission characteristics following passage through different species and strain selection pressures can be exerted by host genetic factors during passage within a species [24], [38], [39], [40], caution is warranted when predicting the risks of CWD transmission from reindeer to other species.

This is the first evidence of CWD transmission to the sub-species *Rangifer tarandus tarandus*, implicating the potential for transmission to others in this genus. Current diagnostic tests, including antemortem RAMALT testing, appear capable of detecting CWD in *Rangifer* species and increased surveillance would be required to determine if natural transmission has indeed occurred. Additional studies are ongoing to chart the distribution of infectivity during the course of disease and determine the influence of *PRNP* polymorphisms in disease susceptibility.

## Materials and Methods

### Ethics Statement

All experimental procedures involving animals were performed under strict accordance with Canadian Council on Animal Care guidelines in such a manner as to minimize suffering. Protocols were approved and monitored by the Animal Care Committee at the Canadian Food Inspection Agency, Ottawa Laboratory – Fallowfield.

### Recipient Animals and Experimental Challenge

The transmissibility of Canadian elk (CWD^ELK^) and white-tailed deer (CWD^WTD^) CWD inocula was investigated in two groups of three, six-month-old reindeer sourced from CWD-free captive herds. Animals were group housed in isolation barns at the Canadian Food Inspection Agency, Ottawa Laboratory – Fallowfield (Ottawa, Ontario).

Inocula were prepared as pooled brain homogenates containing 5 g of brain material in 20 ml of physiological saline. The CWD^ELK^ inoculum contained material from 12 animals in one captive herd while the CWD^WTD^ inoculum was derived from 3 positive animals in a different captive herd and mixing of animals between herds had not knowingly occurred. All source animals were clinically affected by CWD and were confirmed positive by ELISA, western blot and IHC. Reindeer were orally inoculated with two 5 g doses of either elk or white-tailed deer brain homogenate on days 0 and 7 of the trial. Each dose was delivered by syringe to the oropharynx and infected animals were monitored daily for evidence of clinical disease.

### Genotyping

Genotype analysis was conducted on nucleic acid extracted from live animal blood samples using a purification kit following the manufacturer’s protocols (Purelink, Invitrogen). Genomic DNA was used as a template in PCR to amplify the *PRNP* open reading frame using a high fidelity polymerase (Platinum Pfx DNA Polymerase, Invitrogen) and the following primers: forward 5′-GGGCATATGATGCTGACACCCTCTTT and reverse 5′-GAGAAAAATGAGGAAAGAGATGAGGAGG. Reaction conditions were as follows: 94°C for 2 min followed by 35 cycles of denaturation (94°C, 15 sec), annealing (53°C, 30 sec), and extension (68°C, 48 sec). PCR amplicons were gel purified, cloned into a Topo-TA cloning vector (Invitrogen), and sequenced using the T7 primer to obtain sequences from at least 10 clones with the *PRNP* gene. Genotyping of reindeer 1 was conducted on antler velvet using the previously described method [41].

### Ante-mortem and Post-mortem Tissue Collection

At several points during each study animals were sedated with a mixture of ketamine and xylazine for blood collection and biopsy of recto-anal mucosa associated lymphoid tissue (RAMALT). RAMALT biopsies were obtained and evaluated by IHC as previously described [9], [42] and were subjected to testing by commercially available TSE detection kits (TeSeE ELISA and TeSeE Western blot, Bio-Rad Laboratories).

If animals displayed evidence of discomfort, immobility or terminal CWD, or reached specific time points in the study they were euthanized with sodium pentobarbital. A broad range of neural and non-neural tissues from all major organ systems were collected (Table 2) and adjacent tissue samples were frozen at −80°C or fixed in 10% neutral buffered formalin.

### Immunohistochemical and PET Blot Testing

Formalin-fixed tissues were embedded into paraffin wax, sectioned at 5-µm thickness and stained with hematoxylin and eosin or by IHC as previously described [9]. Briefly, following antigen retrieval by autoclaving in citrate buffer solution (DAKO Target Antigen Retrieval), IHC for PrP^CWD^ was performed on an automated immunostainer (Ventana Medical Systems, Tucson, Arizona, USA) using the monoclonal antibody F99/97.6.1 (VMRD, Pullman, Washington, USA) and AEC detection kit (Ventana Medical Systems). Positive and negative staining were differentiated based on comparisons with control tissues included in each run. Paraffin-embedded tissue (PET) blot was conducted as previously described [43], mounting paraffin-embedded sections on a polyvinylidene fluoride (PVDF) membrane (Immobilon-P, Millipore), digesting with Proteinase K (250 µg/ml) and staining with F99/97.6.1 (VMRD, Pullman, Washington, USA).

### ELISA and Western Blot

Fresh or previously frozen unfixed tissue samples were analysed by ELISA and western blot using commercially available TSE detection kits (TeSeE ELISA and TeSeE Western blot, Bio-Rad Laboratories) according to manufacturer’s instructions and as previously described [9]. The ELISA cutoff value was calculated as the average optical density readings of the negative controls plus a fixed value of 0.09 units. Western blots were run with molecular mass markers and CWD positive and negative control elk brain homogenates.

## Acknowledgments

The authors acknowledge the support staff in the Transmissible Spongiform Encephalopathy Unit and the Animal Care Facility at the Ottawa Laboratory Fallowfield for their outstanding technical assistance, in particular Patricia Shaffer, Steven Foster, Dariush Ghazi, Andrei Soutyrine, Antanas Staskevicius and Nishandan Yogasingham. We thank Dr. David Pride for his comments and suggestions, and Olivia Winson and Melanie Lucero for the excellent technical support. Disclaimer: Mention of trade names and commercial products is solely for the purpose of providing information and does not necessarily imply endorsement of their use by the Canadian Food Inspection Agency.

## References

[1] Mathiason CK, Powers JG, Dahmes SJ, Osborn DA, Miller KV (2006). Infectious prions in the saliva and blood of deer with chronic wasting disease.. Science.

[2] Haley NJ, Mathiason CK, Zabel MD, Telling GC, Hoover EA (2009). Detection of sub-clinical CWD infection in conventional test-negative deer long after oral exposure to urine and feces from CWD+ deer.. PLoS One.

[3] Johnson CJ, Pedersen JA, Chappell RJ, McKenzie D, Aiken JM (2007). Oral transmissibility of prion disease is enhanced by binding to soil particles.. PLoS Pathog.

[4] Miller MW, Williams ES, Hobbs NT, Wolfe LL (2004). Environmental sources of prion transmission in mule deer.. Emerg Infect Dis.

[5] Williams ES, Young S (1980). Chronic wasting disease of captive mule deer: a spongiform encephalopathy.. J Wildl Dis.

[6] Kim TY, Shon HJ, Joo YS, Mun UK, Kang KS (2005). Additional cases of Chronic Wasting Disease in imported deer in Korea.. J Vet Med Sci.

[7] Sigurdson CJ (2008). A prion disease of cervids: chronic wasting disease.. Vet Res.

[8] Sigurdson CJ, Aguzzi A (2007). Chronic wasting disease.. Biochim Biophys Acta.

[9] Balachandran A, Harrington NP, Algire J, Soutyrine A, Spraker TR (2010). Experimental oral transmission of chronic wasting disease to red deer (*Cervus elaphus elaphus*): early detection and late stage distribution of protease-resistant prion protein.. Can Vet J.

[10] Spraker TR, Miller MW, Williams ES, Getzy DM, Adrian WJ (1997). Spongiform encephalopathy in free-ranging mule deer (*Odocoileus hemionus*), white-tailed deer (*Odocoileus virginianus*) and Rocky Mountain elk (*Cervus elaphus nelsoni*) in northcentral Colorado.. J Wildl Dis.

[11] Baeten LA, Powers BE, Jewell JE, Spraker TR, Miller MW (2007). A natural case of chronic wasting disease in a free-ranging moose (*Alces alces shirasi*).. J Wildl Dis.

[12] Fox KA, Jewell JE, Williams ES, Miller MW (2006). Patterns of PrPCWD accumulation during the course of chronic wasting disease infection in orally inoculated mule deer (*Odocoileus hemionus*).. J Gen Virol.

[13] Sigurdson CJ, Spraker TR, Miller MW, Oesch B, Hoover EA (2001). PrP(CWD) in the myenteric plexus, vagosympathetic trunk and endocrine glands of deer with chronic wasting disease.. J Gen Virol.

[14] Sigurdson CJ, Williams ES, Miller MW, Spraker TR, O’Rourke KI (1999). Oral transmission and early lymphoid tropism of chronic wasting disease PrPres in mule deer fawns (*Odocoileus hemionus*).. J Gen Virol.

[15] Angers RC, Browning SR, Seward TS, Sigurdson CJ, Miller MW (2006). Prions in skeletal muscles of deer with chronic wasting disease.. Science.

[16] Daus ML, Breyer J, Wagenfuehr K, Wemheuer WM, Thomzig A (2011). Presence and seeding activity of pathological prion protein (PrP(TSE)) in skeletal muscles of white-tailed deer infected with chronic wasting disease.. PLoS One.

[17] Hamir AN, Miller JM, Cutlip RC (2004). Failure to detect prion protein (PrPres) by immunohistochemistry in striated muscle tissues of animals experimentally inoculated with agents of transmissible spongiform encephalopathy.. Vet Pathol.

[18] Spraker TR, Zink RR, Cummings BA, Wild MA, Miller MW (2002). Comparison of histological lesions and immunohistochemical staining of proteinase-resistant prion protein in a naturally occurring spongiform encephalopathy of free-ranging mule deer (*Odocoileus hemionus*) with those of chronic wasting disease of captive mule deer.. Vet Pathol.

[19] Pushie MJ, Shaykhutdinov R, Nazyrova A, Graham C, Vogel HJ (2011). An NMR metabolomics study of elk inoculated with chronic wasting disease.. J Toxicol Environ Health A.

[20] Rhyan JC, Miller MW, Spraker TR, McCollum M, Nol P (2011). Failure of fallow deer (*Dama dama*) to develop chronic wasting disease when exposed to a contaminated environment and infected mule deer (*Odocoileus hemionus*).. J Wildl Dis.

[21] Hamir AN, Greenlee JJ, Nicholson EM, Kunkle RA, Richt JA (2011). Experimental transmission of chronic wasting disease (CWD) from elk and white-tailed deer to fallow deer by intracerebral route: final report.. Can J Vet Res.

[22] Riek R, Hornemann S, Wider G, Billeter M, Glockshuber R (1996). NMR structure of the mouse prion protein domain PrP(121–231).. Nature.

[23] Kurt TD, Telling GC, Zabel MD, Hoover EA (2009). Trans-species amplification of PrP(CWD) and correlation with rigid loop 170 N.. Virology.

[24] Sigurdson CJ, Nilsson KP, Hornemann S, Manco G, Fernandez-Borges N (2010). A molecular switch controls interspecies prion disease transmission in mice.. J Clin Invest.

[25] Apostol MI, Wiltzius JJ, Sawaya MR, Cascio D, Eisenberg D (2011). Atomic structures suggest determinants of transmission barriers in mammalian prion disease.. Biochemistry.

[26] Cronin MA, MacNeil MD, Patton JC (2005). Variation in mitochondrial DNA and microsatellite DNA in caribou (*Rangifer tarandus*) in North America.. Journal of Mammalogy.

[27] Lapointe JM, Leclair D, Mesher C, Balachandran A (2002). Screening for chronic wasting disease in caribou in northern Quebec.. Can Vet J.

[28] Cronin MA, Patton JC, Balmysheva N, MacNeil MD (2003). Genetic variation in caribou and reindeer (*Rangifer tarandus*).. Anim Genet.

[29] Happ GM, Huson HJ, Beckmen KB, Kennedy LJ (2007). Prion protein genes in caribou from Alaska.. J Wildl Dis.

[30] Belay ED, Maddox RA, Williams ES, Miller MW, Gambetti P (2004). Chronic wasting disease and potential transmission to humans.. Emerg Infect Dis.

[31] Mawhinney S, Pape WJ, Forster JE, Anderson CA, Bosque P (2006). Human prion disease and relative risk associated with chronic wasting disease.. Emerg Infect Dis.

[32] Anderson CA, Bosque P, Filley CM, Arciniegas DB, Kleinschmidt-Demasters BK (2007). Colorado surveillance program for chronic wasting disease transmission to humans: lessons from 2 highly suspicious but negative cases.. Arch Neurol.

[33] Sandberg MK, Al-Doujaily H, Sigurdson CJ, Glatzel M, O’Malley C (2010). Chronic wasting disease prions are not transmissible to transgenic mice overexpressing human prion protein.. J Gen Virol.

[34] Kong Q, Huang S, Zou W, Vanegas D, Wang M (2005). Chronic wasting disease of elk: transmissibility to humans examined by transgenic mouse models.. J Neurosci.

[35] Tamguney G, Giles K, Bouzamondo-Bernstein E, Bosque PJ, Miller MW (2006). Transmission of elk and deer prions to transgenic mice.. J Virol.

[36] Marsh RF, Kincaid AE, Bessen RA, Bartz JC (2005). Interspecies transmission of chronic wasting disease prions to squirrel monkeys (*Saimiri sciureus*).. J Virol.

[37] Race B, Meade-White KD, Miller MW, Barbian KD, Rubenstein R (2009). Susceptibilities of nonhuman primates to chronic wasting disease.. Emerg Infect Dis.

[38] Beringue V, Vilotte JL, Laude H (2008). Prion agent diversity and species barrier.. Vet Res.

[39] Collinge J (2010). Medicine. Prion strain mutation and selection.. Science.

[40] Angers RC, Kang HE, Napier D, Browning S, Seward T (2010). Prion strain mutation determined by prion protein conformational compatibility and primary structure.. Science.

[41] O’Rourke KI, Spraker TR, Hamburg LK, Besser TE, Brayton KA (2004). Polymorphisms in the prion precursor functional gene but not the pseudogene are associated with susceptibility to chronic wasting disease in white-tailed deer.. J Gen Virol.

[42] González L, Dagleish MP, Martin S, Dexter G, Steele P (2008). Diagnosis of preclinical scrapie in live sheep by the immunohistochemical examination of rectal biopsies.. Vet Rec.

[43] Schulz-Schaeffer WJ, Tschoke S, Kranefuss N, Drose W, Hause-Reitner D (2000). The paraffin-embedded tissue blot detects PrP(Sc) early in the incubation time in prion diseases.. Am J Pathol.

[44] Wilson GA, Nakada SM, Bollinger TK, Pybus MJ, Merrill EH (2009). Polymorphisms at the PRNP gene influence susceptibility to chronic wasting disease in two species of deer (*Odocoileus Spp.*) in western Canada.. J Toxicol Environ Health A.

[45] Heaton MP, Leymaster KA, Freking BA, Hawk DA, Smith TP (2003). Prion gene sequence variation within diverse groups of U.S. sheep, beef cattle, and deer.. Mamm Genome.

[46] Jewell JE, Conner MM, Wolfe LL, Miller MW, Williams ES (2005). Low frequency of PrP genotype 225SF among free-ranging mule deer (*Odocoileus hemionus*) with chronic wasting disease.. J Gen Virol.

[47] O’Rourke KI, Besser TE, Miller MW, Cline TF, Spraker TR (1999). PrP genotypes of captive and free-ranging Rocky Mountain elk (*Cervus elaphus nelsoni*) with chronic wasting disease.. J Gen Virol.

[48] Brayton KA, O’Rourke KI, Lyda AK, Miller MW, Knowles DP (2004). A processed pseudogene contributes to apparent mule deer prion gene heterogeneity.. Gene.

